# Retraction: Near-cure in patients with Gadolinium deposition disease undergoing intravenous DTPA chelation

**DOI:** 10.3389/ftox.2024.1528757

**Published:** 2024-11-25

**Authors:** Frontiers Editorial Office

The publisher retracts the 25 July 2024 article cited above.

Following publication, the publisher found evidence of multiple undisclosed conflicts of interest that undermined the integrity of the peer review process. As the scientific integrity of the article cannot be guaranteed, and in adherence to the recommendations of the Committee on Publication Ethics (COPE), the article is retracted.

This retraction was approved by the Chief Executive Editor of Frontiers. The authors received a communication regarding the retraction and had a chance to respond. This communication has been recorded by the publisher. The authors do not agree to this retraction.

